# Investigating the effect of group counseling on family stress and anxiety of primiparous mothers during delivery

**DOI:** 10.1186/s13030-019-0148-1

**Published:** 2019-03-26

**Authors:** Fariba Alaem, Amir Jalali, Afshin Almasi, Alireza abdi, Mozhgan khalili

**Affiliations:** 1 Student research committee, Kermanshah University of Medical Sciences, Kermanshah, Iran; 2 Psychiatric Nursing Department, School of Nursing and Midwifery, Substance Abuse Prevention Research Center, Kermanshah University of Medical Sciences, Kermanshah, Iran; 3 Research center of Environmental determinants of Health, Kermanshah University of Medical Sciences, Kermanshah, Iran; 4 Emergency and Critical Care Nursing Department, Nursing and Midwifery School, Kermanshah University of Medical Sciences, Kermanshah, Iran; 5 Midwifery Department, Nursing and Midwifery School, Kermanshah University of Medical Sciences, Kermanshah, Iran

**Keywords:** Stress, Anxiety, Family, Childbirth, Cognitive counseling

## Abstract

**Background:**

Family is considered as the first source of care and support for the mother. Family stress and anxiety can be transmitted to the pregnant mother and have negative effects on pregnancy, childbirth, postpartum, and even their fetus. Given that one of the policies of the World Health Organization is to emphasize “safe family is safe with mother” this study was aimed to determine the effect of group counseling on family stress and anxiety of primiparous mothers during delivery.

**Methods:**

This quasi-experimental study was conducted in 2016 on 72 family members of pregnant women who were referred to midwifery clinics in two health centers in Saveh-Iran. In this research, two members of each family (husband and other family of the pregnant woman) were selected using convenience sampling and were randomly divided into two groups of 36 families. At the beginning of the third trimester of pregnancy, standard questionnaires of Cohen’s Perceived Stress and Cattell Anxiety scale were completed by both groups. For the intervention group, six sessions of cognitive group counseling were held weekly and the control group did not receive the intervention. Then, at the time of hospitalizing pregnant women for childbirth, the questionnaires were completed again by the family of both groups and the two groups were compared in terms of stress and anxiety before and after the intervention. Data were analyzed by SPSS software version 16 using appropriate statistical tests.

**Findings:**

The findings showed that there was no significant difference between mean scores of stress, anxiety, hidden anxiety and obvious anxiety before intervention in both intervention and control groups. In the intervention group, Perceived stress score (*p* < 0.001), anxiety score (*p* < 0.001), hidden anxiety score (*p* = 0.003) and obvious anxiety score (*p* < 0.001) after intervention, with a statistically significant difference were lower than the control group.

**Conclusion:**

Group counseling can reduce the stress and anxiety of the family of primiparous mothers.

**Electronic supplementary material:**

The online version of this article (10.1186/s13030-019-0148-1) contains supplementary material, which is available to authorized users.

## Introduction

Childbirth is a pleasant thing that can have a profound effect on the cohesion of the family. Moreover, family is the first source of care and support for the mother, therefore, one of the important policies of care is to protect the family as caregivers of newly delivered women [1]. The family is the smallest unit of society, which is the source of human emotions and interpersonal interactions, so that the health and prosperity of each society is in the family’s health and development [2]. The physical, emotional and psychological needs of individuals are fulfilled in the family, and family’s function affects the health status of its members. When the function of the family is weaker, the psychological problems of family members will also increase [3]. A husband, as a key member of the family, prepares himself for fatherhood during the pregnancy by supporting his partner and cooperating with her [4]. For this reason, pregnancy is a very important time in order to prepare fathers to help with maternal care, to obtain information about pregnancy and childbirth, and to initiate parental roles and to ask for help from others and health care providers [5].

Although becoming a mother can be enjoyable for parents and family, it can also cause tensions and worries that are due to physical and psychological changes caused during pregnancy and childbirth [6]. Family stress while dealing with new and stressful situations causes discomfort or frustration for family members, and if the family fails to adapt to the new situation, the tension disrupts the family system [1]. Anxiety is a very unpleasant sensation that appears as a sense of intense fear or panic, or doubt about an unknown agent [7]. Obvious anxiety is an acute and variable state of anxiety which varies in different situations, whereas, hidden anxiety is a permanent pattern of anxiety that is known as the temperamental character of an individual [5]. Anxiety is one of the results of stress and is generated in response to an internal or external stimulus and can produce physical, emotional, cognitive or behavioral symptoms [8].

Stress is the external pressure that is exerted on the person, and if it is not controlled and managed, it is likely that the person surrenders. When the external stimulus gets bigger, the probability of resistance decreases. Factors causing stress, stress levels and the response to it vary from person to person and depend on their degree of flexibility [9].The stress and anxiety levels increase in the third trimester of pregnancy as the delivery time approaches so that 90% of stress and anxiety during pregnancy is related to the process of delivery [10, 11]. Stress affects all aspects of human performance. The effect of stress on physiological, cognitive, emotional and behavioral systems is different [12]. Frustration, anger, anxiety, fear, apprehension, and irritability show that stress has affected the individual’s emotional system [12]. Family stress and anxiety can be transmitted to the pregnant mother, increase adrenaline hormone and decrease oxytocin hormone, and can also be followed by the initiation of a defective circle of pain, fear, and muscle stiffness in the pregnant mother and can disrupt the muscular activity of the uterus and prolong delivery [13].

One of the ways that can develop people’s skills against tension and reduce stress and anxiety is group counseling [14]. It seems that through family counseling, family stress and anxiety can be reduced; thereby, it can improve the unpleasant outcomes of pregnancy and childbirth for the mother and the baby [15]. Despite the fact that one of the policies of the World Health Organization is to emphasize “safe family is safe with mother,” no specific studies have been conducted regarding the reduction of the stress and anxiety of the pregnant mother’s family and the implementation of the necessary counseling program in this regard. Therefore, the present study was aimed to determine the effect of group counseling on family stress and anxiety of primiparous mothers during delivery.

## Methods

This research is a quasi-experimental study. The study was compiled by families of primiparous pregnant mothers (the pregnant woman’s husband and the person who would take care of her during childbirth and after delivery, which in most cases was the mother of the pregnant woman) in Saveh-Iran. Due to the lack of a study exactly the same as the present study, based on the expected results of the study of Duncan and Bardcke [16], at 95% confidence level and the 90% test power to detect differences, and considering the probability of dropping samples, the number of samples was calculated at 36 families in each group. It is notable that there are no routine programs for detecting and caring for the anxiety of pregnant women and their family members in the healthcare system of Iran.

Inclusion criteria for the study participants (husband and mother of the pregnant woman) were as follows: first and single pregnancy of the pregnant woman, consent to participate in the study, not a high-risk pregnancy, delivery in one of the hospitals in Saveh-Iran, no mourning in the last three months, the pregnant woman having no medical conditions or surgery, and lack of mental illness and depression (the pregnant woman and her husband and mother), not having drug addiction, being fluent in Persian, no history of participating in childbirth preparation classes, all participants in the study), no history of maternal death for family members, no employment of family members of the participants in the health care field.

Exclusion criteria included: premature delivery before the end of the counseling sessions, pregnancy complications such as pregnancy toxicity, placental abruption, gestational diabetes and any other complications that put the pregnant mother in the high-risk mothers group, becoming a candidate for cesarean delivery, the absence of a participant during the intervention and at the time of delivery, and not attending two or more counseling sessions. The samples were selected using convenience sampling and were randomly assigned to intervention and control groups, based on the cards A (control group) and B (intervention group).

Data collection tools in this study were three questionnaires. The first part was related to the demographic data of the participants in the study. It included information on the age, sex, monthly income, education level, smoking or drug use, a history of the disease or taking medication.

The second part consisted of the Cohen’s Perceived Stress Questionnaire designed by Cohen et al. [17]. The questionnaire has 4, 10, and 14 item versions that are used to measure perceived general stress in the past month. It is also used to measure thoughts and feelings about stressful events, controlling, overcoming, and coping with stress and experienced stresses. Moreover, this scale examines the risk factors for behavioral disorders and shows the process of stressful relations. In this research, the 14-item version was used. It scores each question based on a Likert scale from “4 = never” to “0 = too much”. On this scale, questions 4–7 and 9–13 are scored in reverse order. The lowest score is zero and the highest score is 56. A higher score indicates more perceived stress. Cronbach’s alpha for this scale has been obtained at 0.84–0.85 in three studies [17]. This questionnaire also has been validated in Iran [18] and has been used in several studies about pregnant women in Iran, and its validity and reliability have been confirmed [19, 20]. Moreover, the reliability of this questionnaire has been tested in other research in Iran. The Cronbach’s alpha of this scale in Salehi Ghadri’s research is 0.75 [21] and is 0.80 in a paper by Sepahvand [22].

The third section was the Cattell Anxiety Scale, The questionnaire was developed by Raymond Bernard Cattel [23] as a short questionnaire to measure anxiety. This tool consists of 40 three-option questions and research samples for which one of the options consistent with its status is selected. From the set of scores, the first 20 questions show hidden anxiety, the second 20 questions show obvious anxiety, and their total represents general anxiety. Validity and reliability of this scale have been approved for use in the Iranian population [24].The test has been validated through its repetitive re-implementation, which has always been above 0.70 [25].

Questionnaires were provided without names and codes. Moreover, for each participant, a written consent was obtained which showed their willingness to participate in the study, and individuals were allowed to leave the study whenever they wanted. After receiving a referral from Kermanshah University of Medical Sciences, the researcher referred the mothers to the midwifery clinics of two health care centers in Saveh. Then, the researcher talked about the research goals with the pregnant women’s families who had met the criteria for entering the study. The pregnant woman’s family included her husband, along with a person who would take care of her during childbirth and after delivery, which in most cases was the mother of the pregnant woman. If they were willing to participate in the study, a written consent form was completed by the family. Then, the demographic form, the Cohen Perception Stress Questionnaire, and the Cattell Anxiety scale were provided to the families at the beginning of the third trimester of pregnancy. A phone number was also given to the families and they were requested to inform the researcher in case of pregnancy complications or premature delivery. There was no intervention for control group families. Families in the intervention group were classified according to the time of admission and were divided into six groups, each group consisting of six families. For each group, six consecutive one and a half hour weekly cognitive group counseling sessions were held.

### Intervention

At the beginning of the cognitive group counseling sessions, after welcoming and introducing the members of the group, explanations were given about secrecy, familiarity with the regulations, objectives, and the way the meetings would be held. In these sessions, participants were encouraged to discuss their stress about delivery. A targeted questionnaire was also used to collect information from participants about childbirth, and the right information of most participants was used to correct the inaccurate information. During these sessions, general information about the anatomy and physiology of pregnancy was given to the participants and answers to the concerns and questions of the families were also given by using viewpoints of the participants. During the cognitive sessions, Catharsis was used to encourage people to express their feelings, as was the use of free association skills, to encourage people to come up with whatever they had come to expect. This was helped by open questions and targeted questions. Moreover, they were helped to achieve the correct understanding and to grow and stabilize the changes. In this regard, relaxation techniques, mental imagery and fantasy were used. After completing counseling sessions, an educational booklet was given to the participants and, at the time of referral for delivery, the questionnaires were completed for the second time by the research samples (all the participants). It is notable that the intervention subjects could call the researcher during the research project to ask questions.

For the next visit of a pregnant mother for prenatal care, each group was assigned a different day of the week to prevent the transfer of information and the content of the sessions. Due to the nature of the study, there was no possibility of blinding for the researcher, however, the statistical counselor (the third author) did not know about the grouping or the mode of intervention. It is notable that we gave a booklet and a counseling session at the end of the study to the control group. All sessions are presented in detail in Table 1.

**Table 1:** Cognitive counseling content of each session

**Session 1:** Welcoming and introducing the group members to each other, clarifying team working and confidentiality, familiarity with regulations, declaring purposes and how to prepare the sessions.	
**Session 2: ** Encourage the participants to declare their stress about delivery, fear about possible injuries to mother and infant, high risk interventions, pain, financial problems, affecting changes, and new responsibilities. The participants attended to all subjects worries.	
**Session 3:** Through asking open questions and getting responses, the information of the participants about delivery were taken, then the incorrect information was modified via presenting accurate data to the participants and giving a response to wrong data. In this session we met the worries of subjects by answering the questions and offering some information about the physiology and anatomy of pregnancy.	
**Session 4:** Relaxation methods were educated through asking purposeful questions and	
responding to wrong information	
**Session 5:** Regarding previous sessions, in this stage we tried to stabilize the positive change in mind by giving additional information	
**Session 6:** For reducing stress and anxiety and enhancing the power and calmness, we assisted the participants in supporting the pregnant woman by imaging the feelings of a delivery situation, accompanied by thinking about favorable and desirable events after delivery.	

Data were analyzed using SPSS software version 16 by descriptive and statistical tests such as, Fisher’s exact test(for nominal variables in two groups), independent t-test, paired t-test, and Wilcoxon and Mann-Whitney tests after checking normality of data by Shapiro-Wilk test. The significance level was 0.05.

### Findings

Of the 72 families who were included in the study, two intervention group families were excluded due to being absent from more than two counseling sessions, one family was excluded due to the absence of the husband during the third session, and two families due to premature delivery before the end of the counseling sessions. This left the data of 67 families (31 in the intervention group and 36 in the control group) available for analysis. Demographic characteristics of caregivers of newly delivered women were homogeneous in the two groups (Table 2).

**Table 2:** Distribution of relative and absolute frequency of caregivers of newly delivered women according to demographic variables

Variable		Intervention group	Control group	Total	Test statistic	*P*-value
		Frequency (%)	Frequency (%)	Frequency (%)		
Educational level	Lower than diploma	26 (83.9)	27 (75)	53 (79.1)	0.793	0.279*
	Higher than diploma	5 (16.1)	9 (25)	14 (20.9)		
Income	Less than 1 million Tomans	9 (29)	13 (36.1)	22 (32.8)	0.378	0.363*
	Above 1 million Tomans	22 (71)	23 (63.9)	45 (67.2)		
Participant’s occupation	housewife	26 (83.9)	29 (80.6)	55 (82.1)	0.125	0.489*
	Other	5 (16.1)	7 (19.4)	12 (17.9)		

*Fisher’s exact test

The mean age of caregivers of newly delivered women was 37.5 ± 8.3 years; 38.9 ± 8.33 and 36.4 ± 8.5 years in the intervention and control groups, respectively. Because the age variation was normally distributed (*P* > 0.05) by Shapiro-Wilk Test, we used Independent t-test, which showed no significant difference in terms of age between the intervention and the control groups (*P* = 0.220).

The results also showed the mean age of spouses in this study to be 25.1 ± 4.03 years; 24.9 ± 3.6 and 25.3 ± 4.4 in the intervention and control groups, respectively. Independent t-test showed no significant difference in (*P* = 0.462). Demographic characteristics of the husbands of the newly delivered woman’ caregivers were homogeneous for the two groups of participants (Table 3).

**Table 3:** Distribution of relative and absolute frequency of husbands according to demographic variables

Variable		Intervention group	Control group	Total	Test statistic	*P*-value
		Frequency (%)	Frequency (%)	Frequency (%)		
Educational level	Lower than diploma	18 (58.1)	23 (63.9)	41 (61.2)	0.238	0.406*
	Higher than diploma	13 (41.9)	13 (36.1)	26 (38.8)		
Income	Less than 1 million Tomans	26 (83.9)	26 (72.2)	52 (77.6)	1.301	0.199*
	Above 1 million Tomans	5 (16.1)	10 (27.8)	15 (22.4)		
participant’s occupation	housewife	24 (77.4)	28 (77.8)	52 (77.6)	0.001	0.600*
	Others	7 (22.6)	8 (22.2)	15 (22.4)		

*Fisher’s exact test

The Shapiro-Wilk test showed that the total stress before intervention in the control group (*P* < 0.001) and obvious anxiety in the intervention group (*P* = 0.012) was non-normal (Table 4), so we used the nonparametric tests such as Mann-Whitney U and Wilcoxon test.

**Table 4:** the normality of quantitative variables by Shapiro-Wilk test

Variable	Group	Shapiro-Wilk statistics	*P*-value
Total anxiety before intervention	control	0.982	0.406
	intervention	0.988	0.791
Total anxiety after intervention	control	0.982	0.373
	intervention	0.976	0.274
Hidden anxiety before intervention	control	0.985	0.548
	intervention	0.987	0.760
Hidden anxiety after intervention	control	0.983	0.466
	intervention	0.963	0.058
Obvious anxiety before intervention	Control	0.987	0.656
	intervention	0.985	0.667
Obvious anxiety after intervention	control	0.985	0.558
	intervention	0.949	0.012*
Perceived stress before intervention	control	0.841	< 0.001*
	intervention	0.970	0.135
Perceived stress before intervention	control	0.974	0.143
	intervention	0.983	0.564

*Significant

Comparison of the mean scores of the two groups for perceived stress, hidden anxiety, obvious anxiety, and total anxiety showed no significant difference before the intervention (Table 5).

**Table 5:** Comparison of perceived stress, hidden anxiety, obvious anxiety before and after intervention in the intervention and control groups

Variable / group		Intervention group	Control group	Statistical test
		Mean ± standard deviation	Mean ± standard deviation	
Perceived stress	Before	24.72 ± 5.31	25.98 ± 5.75	*Z* = 1.199 *P* = 0.233
	After	22.14 ± 4.97	26.81 ± 3.49	*t* = 6.19 *P* < 0.001
Statistical test		*t* = 3.68 *P* < 0.001	*Z* = 1.57 *P* = 0.116	
Hidden anxiety,	Before	17.48 ± 3.99	17.66 ± 14.40	*t* = 0.134 *P* = 0.893
	After	15.12 ± 9.50	17.86 ± 4.12	*t* = 3.058 P = 0.003
Statistical test		*t* = 3.25 *P* = 0.002	*t* = 0.643 0.522	
Obvious anxiety	Before	15.11 ± 6.03	16.97 ± 5.25	*t* = 1.53 *P* = 0.126
	After	12.61 ± 5.70	17.25 ± 4.47	*Z* = 4.849 *P* < 0.001
Statistical test		*Z* = 3.65 *P* = 0.001	*t* = 1.044 *P* = 0.30	
Total anxiety	Before	33.14 ± 7.53	34.51 ± 8.63	*t* = 0.970 *P* = 0.334
	After	27.74 ± 9.16	35.02 ± 7.41	*t* = 6.35 *P* < 0.001
Statistical test		*t* = 4.25 *P* < 0.001	*t* = 1.35 0.181	

Comparison of the mean score for perceived stress of the two groups before and after the intervention showed a statistically significant difference, and the mean was reduced after intervention. As Table 5 shows, the mean perceived stress score after intervention changed from 24.72 ± 5.31 to 22.14 ± 4.97 in the intervention group and from 25.98 ± 5.75 to 49.81 ± 3.49 in the control group. Based on paired t-test, the mean was significantly decreased after intervention in the intervention group (*p* < 0.001).

Comparison of the mean scores for hidden anxiety before and after the intervention showed a significant difference and was reduced after the intervention. As Table 5 shows, the mean score for hidden anxiety after intervention changed from 17.48 ± 3.99 to 15.12 ± 9.5 in the intervention group and from 17.66 ± 4.40 to 17.86 ± 4.12 in the control group. Based on paired t-test, the mean score was significantly decreased after the intervention in the intervention group (*p* = 0.002).

Comparison of the mean scores for obvious anxiety before and after the intervention showed a significant difference, decreasing after the intervention. As Table 5 shows, the mean score for obvious anxiety after intervention changed from 15.11 ± 6.03 to 12.61 ± 5.70 in the intervention group and from 16.97 ± 5.25 to 17.25 ± 4.47 in the control group. Based on Wilcoxon test, the mean was significantly decreased after the intervention in the intervention group (*p* = 0.001).

Comparison of the mean total anxiety scores before and after the intervention showed a statistically significant difference, decreasing after the intervention. As Table 5 shows, the mean total anxiety score after intervention changed from 33.14 ± 7.53 to 27.74 ± 9.16 in the intervention group and from 34.51 ± 8.63 to 35.02 ± 7.41 in the control group. Based on paired t-test, the mean score was significantly decreased after intervention in the intervention group (*p* < 0.001).

## Discussion

This study was conducted on the effect of group counseling on family stress and anxiety during the delivery of primiparous mothers. During pregnancy and childbirth, several factors lead to anxiety in the mother, the most common of which is the lack of maternal trust in the cause of labor and the fear of labor pain. These factors are reduced to a great extent by appropriate training and counseling [25].The family of pregnant women, along with them, experience this anxiety and tension. If the family cannot adapt itself to the stressful situation, family health may be disturbed [26]. Counseling can expand people’s skills against tension and reduce stress and anxiety [27]. Increasing the participation of the family and the spouse is possible through providing sufficient training and information to transfer to parenthood, as well as providing facilities for social communication [28].The Husband and relatives of the pregnant woman who are in close contact with her and who support her can play a role in her mental health, This depends on their own mental health, proper knowledge, and right information about pregnancy and childbirth [29].

The findings of this study showed the desired effect of group counseling on perceived stress, hidden anxiety, obvious anxiety, and total anxiety of the family of primiparous mothers. The mean scores for perceived stress, hidden anxiety, obvious anxiety, and total anxiety in the families after intervention were significantly lower than those of the control group. In reviewing the literature, most conducted studies investigated anxiety and stress in mothers during pregnancy and the postpartum period, and a few studies have looked at this issue in the husbands and families of the pregnant women. In this regard, Darwin et al., in 2017, in a study about fathers’ views and experiences of their own mental health during pregnancy, mothers who received counseling and support services along with their husbands during their delivery and afterwards were less stressed and anxious than others [29]. These results are in line with the results of this study. In the pregnancy period, fathers as well as mothers need to have good mental health because fathers are also prone to stress and anxiety during pregnancy, which can affect the health of the mother and the baby. This is while the provided services have focused more on the needs of mothers and there is a lack of information about the experiences and needs of the fathers. This issue emphasizes the importance of counseling the father and other family members during pregnancy [29]. Larissa et al., in 2009, in California examined the effect of mindfulness exercises on the stress and anxiety of pregnant mothers and their husbands. Their study reported that mindfulness exercises were able to reduce the amount of stress and anxiety [30].

In the current study, family members, including the husband of a pregnant woman or a supporter of the pregnant woman were enrolled and six sessions of group counseling were held. Although there were differences from their study in the target group and in how the intervention was performed, pregnant mothers were not included and cognitive counseling was used instead of mindfulness training, in both studies a reduction in stress and family anxiety was reported. In 2017, Backstrom reported in a study regarding partners’ perceptions of professional support during the pregnancy of women, supporting couples was done by providing sufficient information to them, which created the opportunity for them to express their feelings and experiences. Therefore, it could increase fathers’ participation in the care process and improve the couples’ relationships. In contrast, lack of parental involvement in the care process increases the sense of unimportance [31]. If we consider the spouse and family in the pregnant mother’s care process separately, they will not be prepared to support the pregnant mother during or after delivery and this may have adverse effects on the health of the mother and the infant. This emphasizes the role of health team members, especially midwives, in counseling and supporting the pregnant mother’s family.

As mentioned, a number of studies have examined the effects of counseling and counseling techniques on pregnant mothers. Delaram and Soltanpour reported that counseling primiparous women in the third trimester of pregnancy is likely to reduce their anxiety during delivery [32]. Hosseininasab et al. (2010) examined the effect of pregnancy training on women’s anxiety levels and reported that training was able to reduce anxiety levels, so that trained women experienced less anxiety compared to the control group [11].

Khanzadeh et al. stated training in cognitive-behavioral relaxation techniques such as progressive muscle relaxation, breathing exercises, and mental imagery can greatly influence the mental health of individuals by regulating physiological functions of the body and bringing them to rest [33]. This training reduces psychological problems, including anxiety, and it also promotes personal control of impulses, emotions or attitudes. Guardino et al. reported that mindfulness sessions reduced the stress and anxiety level of pregnant mothers [34]. Shobeiri et al. reported that progressive relaxation counseling can be effective in reducing the obvious anxiety of primiparous mothers during pregnancy [35]. The findings of the above-mentioned studies are consistent with the findings of this study, which may be due to the similar methodology.

In the study of Bahrami et al., education during pregnancy led to a significant reduction in the level of anxiety in the postpartum period in the intervention group [36]. Despite differences in the methodology and the subjects, the result was consistent with the results of this study, which confirmed the effect of training and counseling on the reduction of the anxiety level.

Teragea et al. showed that stress management exercises during pregnancy did not significantly change the level of hidden anxiety before and after intervention [37]. The non-alignment of the results of this study in comparison with the present study may be due to a difference in the intervention method used to reduce anxiety.

### Limitation

The limitations of this study include that some family members did not attend counseling sessions from the beginning of the study. The researcher tried to create a quiet environment with maximum cooperation and coordination with the families when holding the counseling sessions and to remind them by phone before the meetings were held, but in the end, in the case of more than two sessions of absence they were excluded from the study. In some cases, the pregnant mother experienced pregnancy complications or the delivery occurred before the consultation ended, limitations which were beyond the control of the researcher. In this case, family members were excluded from the study.

## Conclusion

In explaining the above findings, it can be stated that counseling can facilitate stress reduction in stressful situations during pregnancy, when both the family and the pregnant mother have high levels of stress and anxiety. Given that a limited number of studies have examined the effect of counseling on the stress and anxiety of the spouse and family of pregnant women and considering that so far such a study has not been published Iran, the present study was aimed to determine the impact of counseling on family stress and anxiety during delivery of primiparous mothers.

It is suggested that future study of the effect of family counseling on the prognosis of childbirth, such as postpartum depression in pregnant women, should be measured. Moreover, the direct effect of family counseling on the level of stress and anxiety of pregnant women during childbirth can be examined.

## Additional file

Additional file 1: Supplementary data file. (SAV 35 kb)
